# Supplementary material for: Large domains of heterochromatin direct the formation of short mitotic chromosome loops
Source: eLife. 2020 Sep 11;9:e57212. doi: 10.7554/eLife.57212 (PMC7515631; doi:10.7554/eLife.57212)
Supplement: Transparent reporting form [file elife-57212-transrepform.docx]

***eLife’s* transparent reporting form**

We encourage authors to provide detailed information *within their submission* to facilitate the interpretation and replication of experiments. Authors can upload supporting documentation to indicate the use of appropriate reporting guidelines for health-related research (see [EQUATOR Network](http://www.equator-network.org/%20)), life science research (see the [BioSharing Information Resource](https://biosharing.org/)), or the [ARRIVE guidelines](http://www.plosbiology.org/article/info:doi/10.1371/journal.pbio.1000412) for reporting work involving animal research. Where applicable, authors should refer to any relevant reporting standards documents in this form.

If you have any questions, please consult our Journal Policies and/or contact us: [editorial@elifesciences.org](mailto:editorial@elifesciences.org).

**Sample-size estimation**

- You should state whether an appropriate sample size was computed when the study was being designed
- You should state the statistical method of sample size computation and any required assumptions
- If no explicit power analysis was used, you should describe how you decided what sample (replicate) size (number) to use

Please outline where this information can be found within the submission (e.g., sections or figure legends), or explain why this information doesn’t apply to your submission:

Sample size for microscopy experiments is indicated in the figure legends and figures themselves for figures 1B, 1E, 1F, 2D, 2E, 3D, 3E, 4B, 4D, 5G-L, 6F and 6G as well as Figure 1 – figure supplement 1E&F ; Figure 3 – figure supplement 1C&D and Figure 5 – figure supplement 1 D-F & J-L. In each case sample size was the maximum number of chromosome spreads that could be clearly imaged for each experiment.

**Replicates**

- You should report how often each experiment was performed
- You should include a definition of biological versus technical replication
- The data obtained should be provided and sufficient information should be provided to indicate the number of independent biological and/or technical replicates
- If you encountered any outliers, you should describe how these were handled
- Criteria for exclusion/inclusion of data should be clearly stated
- High-throughput sequence data should be uploaded before submission, with a private link for reviewers provided (these are available from both GEO and ArrayExpress)

Please outline where this information can be found within the submission (e.g., sections or figure legends), or explain why this information doesn’t apply to your submission:

Figure legends specify that three independent repeats were performed for all ChIP experiments in Figures 2A, 3G-I, 4E, 5M-R and Figure 2 – figure supplement 1.

**Statistical reporting**

- Statistical analysis methods should be described and justified
- Raw data should be presented in figures whenever informative to do so (typically when N per group is less than 10)
- For each experiment, you should identify the statistical tests used, exact values of N, definitions of center, methods of multiple test correction, and dispersion and precision measures (e.g., mean, median, SD, SEM, confidence intervals; and, for the major substantive results, a measure of effect size (e.g., Pearson's r, Cohen's d)
- Report exact p-values wherever possible alongside the summary statistics and 95% confidence intervals. These should be reported for all key questions and not only when the p-value is less than 0.05.

Please outline where this information can be found within the submission (e.g., sections or figure legends), or explain why this information doesn’t apply to your submission:

Statistical tests performed on the data are described in the appropriate figure legends.

For chromosome profile graphs from microscopy signal intensity measurements, data points represent averages, in some cases normalized, with error bars representing +/- SEM, as indicated. Data distribution at different points was compared by the KS test, with p-values indicated in each case (Figures 1B, 1E, 1F, 2D, 2E, 3D, 3E, 4B, 4D, 5G-L, 6F-I and Figure 1 – figure supplement 1E&F, Figure 3 – figure supplement 1C&D, Figure 5 – figure supplement 1 D-F & J-L, Figure 6 – figure supplement 2).

For ChIP bar graphs, bars represent average enrichment normalized to positive control, with error bars representing +/- SEM, as indicated. Data were compared between each other by the t-test as described, with the indicated p-values (Figures 2A, 3G-I, 4E, 5M-R and Figure 2 – figure supplement 1).

(For large datasets, or papers with a very large number of statistical tests, you may upload a single table file with tests, Ns, etc., with reference to sections in the manuscript.)

**Group allocation**

- Indicate how samples were allocated into experimental groups (in the case of clinical studies, please specify allocation to treatment method); if randomization was used, please also state if restricted randomization was applied
- Indicate if masking was used during group allocation, data collection and/or data analysis

Please outline where this information can be found within the submission (e.g., sections or figure legends), or explain why this information doesn’t apply to your submission:

Group allocation is not relevant to the experiments performed in our submission.

**Additional data files (“source data”)**

- We encourage you to upload relevant additional data files, such as numerical data that are represented as a graph in a figure, or as a summary table
- Where provided, these should be in the most useful format, and they can be uploaded as “Source data” files linked to a main figure or table
- Include model definition files including the full list of parameters used
- Include code used for data analysis (e.g., R, MatLab)
- Avoid stating that data files are “available upon request”

Please indicate the figures or tables for which source data files have been provided:

Data excel files have been provided for chromosome profile graphs and ChIP bar graphs in Figures 1B, 1E, 1F, 2A, 2D, 2E, 3D-I, 4B, 4D, 4E, 5G-R, 6F and 6G as well as Figure 1 – figure supplement 1E&F, Figure 2 – figure supplement 1, Figure 3 – figure supplement 1C&D, Figure 5 – figure supplement 1D-F&J-L.

DNA sequencing and nanopore data were uploaded to the Sequence Read Archive with project ID PRJNA629899. Hi-C data was uploaded to GEO with accession ID GSE149677. It can be viewed at  <https://www.ncbi.nlm.nih.gov/geo/query/acc.cgi?acc=GSE149677> with the code “gturomaaxfcphcl”.
